# The multifaceted roles of ZIC genes in cancer: from development regulators to cancer modulators

**DOI:** 10.1007/s12672-025-03511-1

**Published:** 2025-09-15

**Authors:** Dina Hesham, Shahenda El-Naggar

**Affiliations:** 1 Tumor Biology Research Program, Basic Research Unit, Research Department, Children’s Cancer Hospital Egypt 57357, 1 Sekket El Emam, El Madbah El Kadeem Yard, Sayeda Zeinab, Cairo, Egypt; 2 Microbiology, Immunology and Biotechnology Department, Faculty of Pharmacy and Biotechnology, German University in Cairo (GUC), Cairo, Egypt

**Keywords:** ZIC genes, Zinc finger, Transcription factors, Development, Cancer, Epigenetic regulation

## Abstract

ZIC genes, which endcode zinc finger transcription factors, are recognized for their foundational roles in vertebrate development and have been increasingly been implicated in various aspects of cancer biology. Initially identified for their critical contributions to cerebellum development and neural patterning, ZIC genes have been found to influence a wide range of cellular processes. This review describes the structural attributes of ZIC genes, their biological functions during development, and their roles in cancer pathogenesis. Recent findings highlight the possibility of a dual nature of ZIC genes in oncogenesis, harboring oncogene or tumor suppressor activities depending on the cancer type and cellular context. We also explored the impact of epigenetic modifications and expression alterations of ZIC genes on tumor behavior, detailing their involvement in key oncogenic pathways including the sonic hedgehog, Wnt/β-Catenin, TGF-β, PI3K/AKT, and MAPK pathways. In conclusion, we aim to provide a better understanding of their complex roles in cancer, opening avenues for targeted therapeutic strategies and advancing diagnostic and prognostic evaluations across various cancers.

## Background

The zinc finger of the cerebellum (ZIC) family genes encode a group of transcription factors central to many developmental processes and cellular functions. These genes are identified by their distinctive zinc finger motifs, a structural feature essential for their role in DNA binding and transcriptional regulation [1–3]. Originally discovered due to their crucial involvement in the development of the cerebellum in vertebrates, the scope of ZIC genes has broadened to include pivotal roles in gastrulation, neural tube closure, and limb patterning, reflecting their widespread influence on embryonic development [4, 5]. In addition, they were shown to play crucial roles in various stages of late brain development, including neuronal migration and axon guidance, which are vital for establishing correct wiring and connectivity within the brain, ensuring that neural circuits are properly formed and functional [6].

Beyond their foundational roles in development, ZIC genes have emerged as significant players in the pathology of various cancers. Alterations in their epigenetic regulation and expression are increasingly associated with the development and progression of malignancies, influencing tumor behavior and patient outcomes [7]. According to data from cBioPortal, ZIC genes harbor copy number alterations in multiple cancer types, especially homozygous deletions in pediatric leukemias and Wilms' tumor [8]. In addition, ZIC2 was reported to be an oncogene in ONGene database [9], while ZIC1 was listed as a tumor suppressor in TSGene database [10]. However, none of the ZIC genes are currently listed in the COSMIC -Cancer Gene Census-, which underscores the need for further functional and genomic validation to confirm their roles as canonical oncogenes or tumor suppressor genes. These genes have been demonstrated to regulate critical oncogenic processes, including cell cycle regulation, evasion of apoptosis , and metastasis, making them key subjects of cancer research [11–13]. For instance, overexpression of specific ZIC genes has been linked to the promotion of oncogenic pathways in meningiomas [14]. In contrast, reduced expression of ZIC genes—frequently through promoter hypermethylation—has been associated with malignancy in cancers such as breast and ovarian cancer [15–17]. While these observations support a tumor-inhibitory role under certain conditions, further mechanistic studies are needed to confirm whether these genes fulfill the classical definitions of tumor suppressors or oncogenes.

The context-dependent, dual functionality of ZIC genes—where they may act as oncogenes in one tissue and tumor-inhibiting factors in another—renders them intriguing targets for therapeutic intervention. Their dysregulation often results from epigenetic alterations, which are potentially reversible, making them amenable to targeted therapies such as DNA demethylating agents or histone modification inhibitors as well as RNA-based therapeutics. Moreover, ZIC genes modulate key signaling pathways such as Wnt/β-catenin and Hedgehog pathways, offering multiple potential points for therapeutic modulation.

This review aims to describe the structural characteristics of ZIC genes, their biological roles in normal development, and epigenetic and expression alterations that influence their function in cancer. In addition, by highlighting how ZIC genes are involved in various cancer pathways and evaluating their potential as diagnostic and prognostic markers, we aim to provide a detailed overview of their significance in advancing the field of oncology. The potential of ZIC genes as diagnostic and prognostic markers holds promise for the future of cancer research and treatment. Understanding the expression patterns and regulatory mechanisms of ZIC genes in cancer is vital for developing targeted therapeutic strategies and improving the diagnostic and prognostic assessments in cancer.

## The functional domains of ZIC proteins

ZIC genes are the vertebrate homologues of the Drosophila gene “odd-paired” (opa), and vertebrate genomes typically code for a family of five genes [18]. In vertebrates, they play a key role in neural and skeletal patterning, while in other species, such as urochordates and protostomes, they contribute to cell fate determination and segmentation. The functions of ZIC genes vary across phylogenetic lines, but their involvement in neural development appears to be conserved, with evidence suggesting that they may have contributed to the unique body plans of different animal groups [1]. The human ZIC gene family comprises of five members, *ZIC1*,* ZIC2*,* ZIC3*,* ZIC4*, and *ZIC5* These genes encode transcription factors involved in regulating genes and are distinguished by their signature C2H2-type zinc finger motifs [19].

**Fig. 1:**
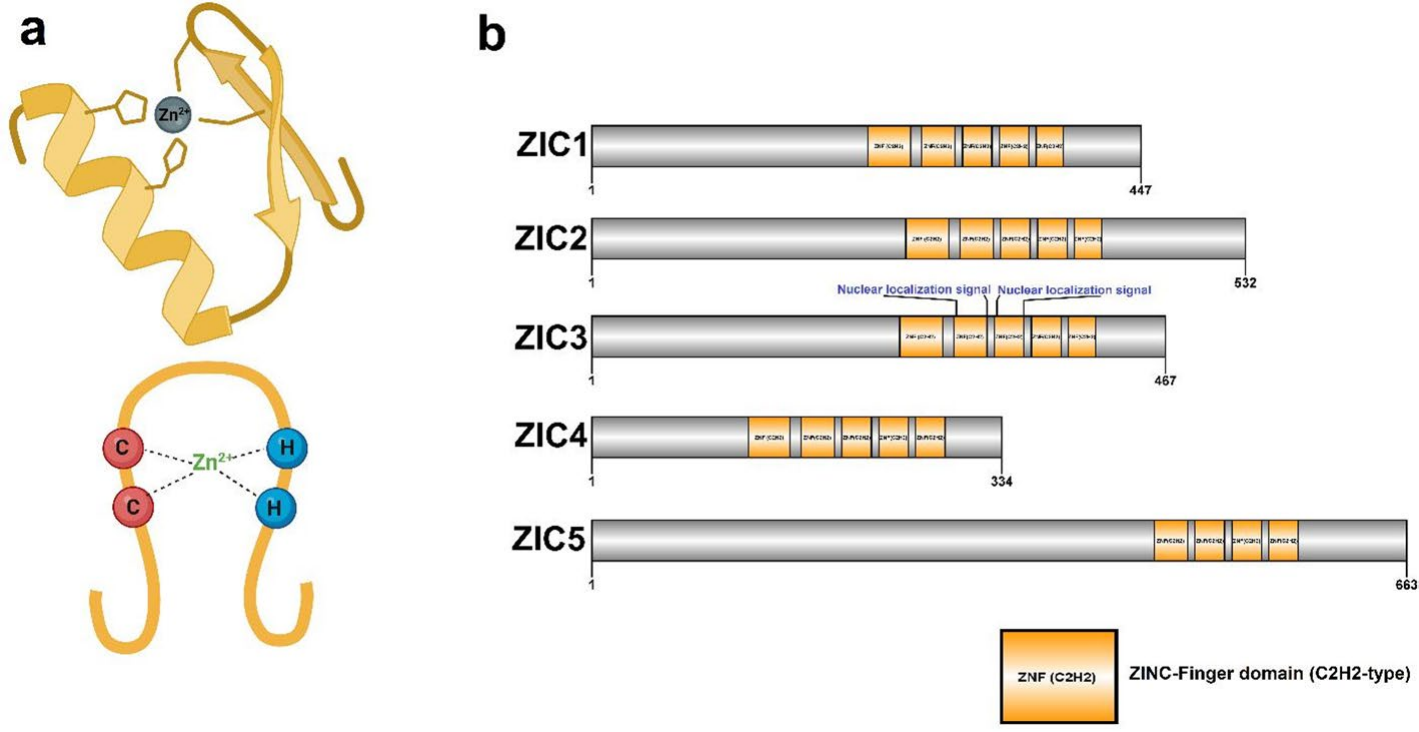
Zinc finger domain and ZIC proteins structure. (a) A zinc finger (ZNF) domain is composed of conserved arrangements of cysteines (C) and histidines (H), which are coordinated by a central zinc ion, resulting in a finger-like structure (C2H2-type zinc fingers). (b) Schematic diagram of ZIC proteins structures showing the zinc finger domain encompassing tandem C2H2-type zinc fingers that are essential for DNA binding and protein-protein interactions. Only ZIC3 has a distinct nuclear localization signals

Generally, a zinc finger (ZNF) motif is composed of approximately 30 amino acid sequences with conserved arrangements of cysteines (C) and histidines (H). A central zinc ion coordinates the cysteine and histidine residues, resulting in a finger-like structure. ZIC genes have a C2H2 arrangement within the ZNF motif (Fig. 1a) [20]. Each ZIC protein typically features five zinc finger motifs However, variability influences DNA binding specificity and transcriptional activity (Fig. 1b). ZIC3 has a specific nuclear localization signal (NLS), which is critical for its function as a transcription factor, guiding the protein into the nucleus via interactions with importin proteins [21]. This NLS, rich in basic amino acids and integrated within the zinc finger domain, ensures efficient recognition and transport through nuclear pore complexes [22].

**Fig. 2:**
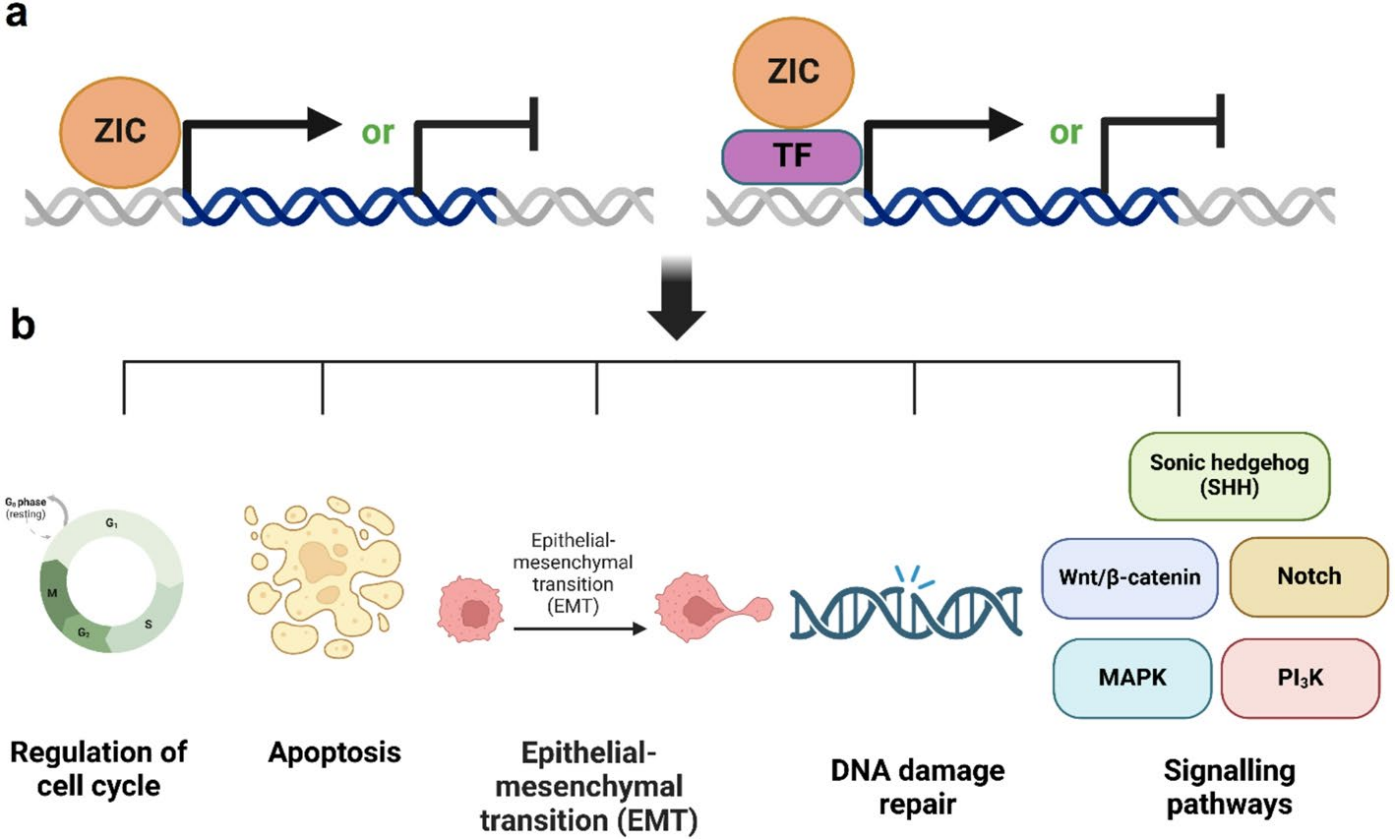
ZIC family genes as transcription factors or co-factors are involved in different processes and signaling pathways. **a** ZIC proteins can act as transcription factors themselves by binding to DNA or by interacting with other proteins (as co-factors) to activate or inhibit the expression of downstream targets. **b** A schematic illustration to show how ZIC proteins function to regulate gene expression, impacting various cellular processes and signaling pathways

The zinc finger motif folds into a ββα structure upon coordinating a zinc ion. The α-helix of this structure is the primary DNA-binding element. Each zinc finger typically recognizes 3–4 bases of DNA, and multiple zinc fingers can be strung together within a single protein to recognize longer DNA sequences, allowing for high specificity in target gene recognition. The side chains of specific amino acids within the α-helix make direct contact with the major groove of the DNA, facilitating specific base pair recognition [23]. These zinc finger domains are also involved in protein-protein interactions [24, 25]. Not only do they act as a transcription factor themselves, ZIC proteins usually interact with other transcription factors to co-regulate target genes (Fig. 2a) [26]. For example, ZIC and Gli proteins were shown to physically and functionally interact, mainly to regulate the Hedgehog signaling pathway. It was revealed that ZIC proteins can facilitate the nuclear translocation of Gli proteins. Furthermore, ZIC proteins can enhance or inhibit the transcriptional activity of GLI proteins depending on the cellular context and the presence of other cofactors [27, 28]. Altogether, this suggests that ZIC proteins possess a complex regulatory capacity that extends beyond simple DNA binding, rendering ZIC genes to be involved in a myriad of cellular processes and modulating vital developmental signaling pathways such as Wnt/β-Catenin, sonic hedgehog (SHH), and notch (Fig. 2b) [28–34].

## ZIC genes are involved in embryonic development and cell differentiation

ZIC genes are foundational role in orchestrating embryonic development. ZIC1 is particularly noted for its role in cerebellar development, influencing cell lineage decisions and patterning within the developing brain. Disruption in ZIC1 expression can lead to cerebellar malformations and neurodevelopmental disorders, highlighting its critical developmental role [35]. ZIC1 and ZIC4 have been demonstrated to play roles during cerebellar development, and multiple developmental alterations are associated with their loss, leading to the Dandy-Walker malformation cerebellar pathogenesis [36]. ZIC2, another prominent family member, plays a significant role in neural tube closure, a process essential for the correct formation of the central nervous system [37]. Mutations or misregulations in ZIC2 are associated with severe birth defects, including holoprosencephaly, in which the brain fails to divide properly into distinct hemispheres [31]. Within neurogenesis, ZIC genes regulate the proliferation and differentiation of neural progenitors [19, 32, 38]. ZIC3, for example, regulates neuronal progenitor proliferation, thereby influencing the fate of neuronal cells during development [39].

## Expression of ZIC genes in cancer

Along with their roles in developmental processes, the role of ZIC family members has been recognized in the pathology of cancer. The alterations in the expression of ZIC genes have been observed across various tumor types, influencing tumor behavior, progression, and response to therapy [40, 41]. For example, ZIC2 showed increased expression and was associated with tumorigenesis and poor outcome in a wide range of cancer types like meningiomas, pancreatic cancer, nasopharyngeal carcinoma, non-small cell lung cancer, neuroblastoma, and gastric cancer. This finding opens up the potential for ZIC2 to be a promising diagnostic and prognostic marker (See Table 1) [42]. On the other hand, ZIC2 was demonstrated to repress tumor growth in breast cancer, and its reduced expression was correlated with short survival [43].

**Table 1:** Expression of ZIC genes in cancer and affected pathways/processes

Gene	Disease	Status	Pathway involved	Publication	Year
ZIC1	Meningiomas	Up-regulated		PMID: 20,199,689	2010
	Liposarcoma	Up-regulated		PMID: 20,713,527	2010
	Colorectal cancer	Methylated (down-regulated)	MAPK and PI3K/Akt pathways, Bcl-xl/Bad/Caspase3 cascade	PMID: 21,347,233	2011
	Ovarian cancer	Methylated (down-regulated)	Sonic hedgehog pathway	PMID: 23,774,800	2013
	Pleural Mesothelioma	Methylated (down-regulated)		PMID: 24,457,242	2013
	Thyroid cancer	Methylated (down-regulated)	PI3K/Akt and MAPK signaling pathways	PMID: 24,684,457	2014
	Endometrial cancer	Up-regulated		PMID: 26,670,443	2015
	Breast cancer	Down-regulated	Akt/mTOR/P70S6K signaling pathway	PMID: 29,956,756	2018
	Gastric cancer	Methylated (down-regulated)	Wnt/β-catenin signaling, Epithelial-mesenchymal transition (EMT), MAPK and PI3K/Akt pathways	PMID: 19,135,984, PMID: 22,799,764, PMID: 26,207,911, PMID: 27,177,248, PMID: 31,909,528	2009, 2012, 2015, 2016, 2020
	Head and neck carcinoma	Methylated (down-regulated)		PMID: 27,553,089	2016
ZIC2	Meningiomas	Up-regulated		PMID: 20,199,689	2010
	Pancreatic cancer	Up-regulated		PMID: 26,318,045	2015
	Liver cancer stem cells (CSCs)	Up-regulated		PMID: 26,426,078	2015
	Nasopharyngeal carcinoma	Up-regulated	Pro-tumor macrophage polarization by activating the JUNB/MCSF axis	PMID: 31,949,842, PMID: 37,479,694	2018, 2023
	Non-small cell lung cancer	Up-regulated	Src/FAK signaling	PMID: 34,514,099	2021
	Colon cancer	Up-regulated	Wnt/β-catenin pathway	PMID: 34,099,631	2021
	Neuroblastoma	Up-regulated		PMID: 35,969,939	2022
	Clear cell renal cell carcinoma	Up-regulated	UBE2C/mTOR signaling pathway	PMID: 37,496,990	2023
	Gastric cancer	Up-regulated	Wnt/β-catenin pathway	PMID: 37,774,522	2023
	Colorectal cancer	Nuclear translocation/high expression	hedgehog signaling/TGF-β signaling pathway	PMID: 31,929,751, PMID: 35,390,314	2020, 2022
	Breast cancer	Down-regulated	JAK/STAT3 pathway	PMID: 32,064,600	2020
ZIC3	Lung cancer	Up-regulated		PMID: 26,498,524	2015
	Breast cancer	Low-expression		PMID: 32,390,766	2020
ZIC4	Medulloblastoma	Up-regulated	Sonic hedgehog pathway	PMID: 20,199,689	2010
	Bladder cancer	Methylated (down-regulated)		PMID: 22,284,968	2012
	Ovarian cancer	Methylated (down-regulated)		PMID: 23,774,800	2013
	Head and neck carcinoma	Methylated (down-regulated)		PMID: 24,786,473, PMID: 27,553,089	2014, 2016
	Liver cancer	Methylated (down-regulated)	FAK and STAT3 signaling	PMID: 33,097,694	2020
	Choroid plexus tumors (pediatric)	Methylated (down-regulated)		PMID: 39,266,576	2024
ZIC5	Meningiomas	Up-regulated	CDK1/CDC25c signaling/ glucose metabolism	PMID: 20,199,689	2010
	Melanoma	Up-regulated		PMID: 27,671,679	2017
	Colorectal cancer	Up-regulated		PMID: 31,392,276, PMID: 33,747,280	2019, 2021
	Prostate cancer	Up-regulated		PMID: 36,127,329	2022
	Pancreatic ductal adenocarcinoma/cholangiocarcinoma	Up-regulated	Wnt/β-catenin pathway	PMID: 35,669,984	2022
	Cervical squamous cell carcinoma	Up-regulated	Increase the expression of CCNB1/CDK1 cell cycle players	PMID: 36,495,760	2023
	Hepatocellular carcinoma	Up-regulated		PMID: 30,086,882, PMID: 35,837,163	2018, 2022
	Non-small cell lung cancer	Up-regulated		PMID: 27,663,664	2016

Aberrant expression of ZIC genes can also result from epigenetic modifications, often leading to their suppression and promotion of tumorigenic processes. ZIC1 and ZIC4 genes are frequently subject to epigenetic silencing through DNA hypermethylation at their promoter regions in several cancers (See Table 1). In ovarian cancer (OC), silencing of ZIC1 and ZIC4 hypermethylation was associated with a poor prognosis, and their repression was correlated with increased proliferation, migration, and invasion in a panel of OC cell lines [15]. ZIC genes can also be subjected to epigenetic silencing by histone methylation. In desmoid tumors, the downregulation of ZIC1 was associated with increased methylation of histone H3 on lysine 9 (H3K9me2) at its promoter region [44]. Additionally, epigenetic silencing of ZIC4 by enhancer of zeste homolog 2 (EZH2) mediated H3K27 trimethylation (H3K27me3) was described in hepatocellular carcinoma (HCC). EZH2 knockdown or EZH2 inhibitor (DZNep) reduced the levels of EZH2 and H3K27me3 at the ZIC4 promoter region, leading to the upregulation of ZIC4 and a reduction in the proliferation and invasiveness of HCC cells [45]. Also, methylation profiling of pediatric choroid plexus tumors (CPTs) categorized different pathological subtypes into two molecular groups, with either favorable outcomes (group A) or poor outcomes (group B). Notably, ZIC4 identified as one of the top differentially methylated genes between the two groups, exhibiting more hypermethylated patterns in group B compared to group A [46, 47]. These findings underscore the potential of ZIC genes as therapeutic targets.

## ZIC genes and their involvement in diverse cancer pathways

ZIC genes are integral to several critical signaling pathways that regulate key cellular processes such as proliferation, differentiation, apoptosis, and migration. Their modulation of these pathways can significantly influence cancer progression and therapy resistance.

### Sonic hedgehog pathway

Sonic Hedgehog is a conserved signaling pathway, is involved in various embryogenesis processes including neurogenesis, maintenance of physiological stem cells,tissue maintenance, regeneration, and normal homeostasis [48, 49]. Misregulation of the SHH pathway has been associated with several malignant transformations and plays an essential role maintenaing of cancer stem cells (CSCs), especially those of epithelial origin [50, 51]. ZIC1 and ZIC4 are known to be regulators of the SHH signaling pathway by antagonizing the transcriptional activity of Gli proteins, and arefound to be silenced by hypermethylation in several types of cancers [15, 52]. In contrast, brain-expressed X-linked 2 (BEX2) protein displayed a negative modulation of SHH signaling by retaining ZIC2 in the cytoplasm and inhibiting its nuclear translocation in colorectal cancer cells, hence inhibiting their migration and metastasis [53].

### Wnt/β-catenin pathway

The Wnt/β-catenin pathway, also known as the canonical Wnt pathway, is well recognized in developmental biology and is essential for maintaining stem cell properties, as well as for carcinogenesis [54]. ZIC genes were shown to be involved in the regulation of the Wnt/β-catenin pathway in different tumors. ZIC2 was demonstrated to activate Wnt/β-catenin signaling by Zic2 directly interacting with β-catenin and by transcriptionally repressing Axin2 expression and consequently promoting the accumulation and nuclear translocation of β-catenin in colon cancer [55]. In gastric cancer, ZIC2 could enhance Wnt signaling by increasing the expression of β-catenin, c-Myc, and MMP-7, while repressing Axin expression [56]. Similarly, ZIC5 increased the expression of β-catenin and Cyclin D1 in the Wnt/β-catenin pathway, leading to enhanced invasion and metastasis of hepatocellular carcinoma [57]. On the contrary, ZIC1 was shown to repress Wnt/β-catenin signaling by repressing Wnt targets such as c-Myc and Cyclin D1 and by interacting with and inhibiting the function of the β-catenin/TCF4 complex in gastric cancer cells [13].

### TGF-β signaling pathway

The transforming growth factor β (TGF-β) family of cytokines is essential for embryonic development, tissue homeostasis, and injury repair by regulating cell proliferation, phenotypic plasticity, migration, metabolic adaptation, and immune surveillance in different cell types [58]. Alterations in TGF-β signaling, especially within epithelial cells, tissue fibroblasts, and immune cells, can disrupt immune tolerance and induce inflammation, hence playing a crucial role in carcinogenesis [59, 60]. In colorectal cancer, ZIC2 modulates TGF-β signaling by inducing TGF-β1 expression and increaings SMAD3 phosphorylation leading to disease progression and metastasis [61].

### PI3K/AKT and MAPK pathway signaling pathway

The phosphoinositide 3-kinase (PI3K)/Akt and mitogen-activated protein kinase (MAPK) signaling pathways are reported to play vital roles in normal cell growth and neuroprotection, via stimulating mitosis, promoting cell proliferation, as well as modulating apoptosis [31, 62, 63]. These pathways also have a significant impact on oncogenesis by supporting the survival and proliferation of cancer cells, including leukemia and various solid tumors [64–66]. In several cancers like thyroid, gastric, and colorectal cancers, ZIC1 was found to be silenced by methylation, and upon overexpression in cancer cells, it was shown to inhibit cell proliferation, induce cell cycle arrest, and apoptosis by blocking the activities of the PI3K/Akt and MAPK pathways [11, 67, 68].

## Discussion

The study of ZIC genes has significantly advanced our understanding of cancer biology, revealingthe complex roles these genes play in promoting and inhibiting tumor growth. As transcription factors are pivotal in developmental processes, ZIC genes are uniquely positioned at the intersection of developmental biology and oncology. Their involvement in essential signaling pathways such as Wnt/β-catenin, SHH, Notch, PI3K/AKT, and MAPK underscores their importance in regulating cellular proliferation, differentiation, apoptosis, and migration, key processes that are often deregulated in cancer.

The diagnostic and prognostic significance of ZIC genes in cancer is profound. As biomarkers, the expression levels of ZIC genes were shown to distinguish between normal and malignant tissues. ZIC5, for instance, is upregulated in several malignancies, suggesting its potential as a diagnostic marker in those types of cancers (See Table 1). Similarly, the epigenetic status of ZIC genes, particularly their promoter methylation patterns, provides insight into diagnosis, as seen with the hypermethylation of ZIC1 and ZIC4 in ovarian and head and neck cancers. Prognostically, ZIC genes are valuable for predicting patient outcomes. High expression levels of certain ZIC genes are associated with poor prognosis and more aggressive cancer phenotypes, as observed with ZIC2 in clear cell renal cell carcinoma [69] and ZIC5 in hepatocellular carcinoma [57]. Hence, their altered expression and epigenetic modifications provide valuable biomarkers for early cancer detection and prognosis. Therapeutically, ZIC genes might offer promising targets for novel cancer treatments. Understanding how ZIC genes affect oncogenic pathways not only provides insights into tumor biology but also opens avenues for developing targeted therapies that could manipulate these genes for clinical benefit by either inhibiting their oncogenic functions or enhancing their tumor suppressor activities [70, 71]. In conclusion, investigating ZIC genes represents a frontier in cancer research. The continued exploration of their roles in cancer will deepen our understanding of tumor biology and pave the way for innovative therapeutic strategies that could improve current treatments.

## Data Availability

No datasets were generated or analysed during the current study.

## Abbreviations

ZIC: Zinc finger of the cerebellum
OPA: Odd-paired
ZNF: Zinc finger
NLS: Nuclear localization signal
SHH: Sonic hedgehog
OC: Ovarian cancer
EZH2: Enhancer of zeste homolog 2
H3K27me3: H3K27 trimethylation
HCC: Hepatocellular carcinoma
CPTs: Choroid plexus tumors
BEX2: Brain-expressed X-linked 2
TGF-β: Transforming growth factorβ
PI3K: Phosphoinositide 3-kinase
CSCs: Liver cancer stem cells
EMT: Epithelial-mesenchymal transition
